# Bacteriophage-host interactions in *Streptococcus thermophilus* and their impact on co-evolutionary processes

**DOI:** 10.1093/femsre/fuad032

**Published:** 2023-06-20

**Authors:** Katherine Lavelle, Brian McDonnell, Gerald Fitzgerald, Douwe van Sinderen, Jennifer Mahony

**Affiliations:** APC Microbiome Ireland and School of Microbiology, University College Cork, Cork, T12 YN60, Ireland; APC Microbiome Ireland and School of Microbiology, University College Cork, Cork, T12 YN60, Ireland; APC Microbiome Ireland and School of Microbiology, University College Cork, Cork, T12 YN60, Ireland; APC Microbiome Ireland and School of Microbiology, University College Cork, Cork, T12 YN60, Ireland; APC Microbiome Ireland and School of Microbiology, University College Cork, Cork, T12 YN60, Ireland

**Keywords:** Food fermentations, phage-resistance, polysaccharide, receptor, adhesion

## Abstract

Bacteriophages (or phages) represent a persistent threat to the success and reliability of food fermentation processes. Recent reports of phages that infect *Streptococcus thermophilus* have highlighted the diversification of phages of this species. Phages of *S. thermophilus* typically exhibit a narrow range, a feature that is suggestive of diverse receptor moieties being presented on the cell surface of the host. Cell wall polysaccharides, including rhamnose-glucose polysaccharides and exopolysaccharides have been implicated as being involved in the initial interactions with several phages of this species. Following internalization of the phage genome, the host presents several defences, including CRISPR-Cas and restriction and modification systems to limit phage proliferation. This review provides a current and holistic view of the interactions of phages and their *S. thermophilus* host cells and how this has influenced the diversity and evolution of both entities.

## Teaser

This review provides an overview of the current knowledge of interactions and co-evolution of Streptococcus thermophilus and its infecting phages.

## Introduction

*Streptococcus thermophilus* is a lactic acid bacterium that has long been associated with the production of fermented dairy products, including yoghurt and Italian- and Swiss-style cheeses. A total of 180 species of streptococci are currently defined (https://lpsn.dsmz.de/genus/streptococcus November 2022), and among these *S. thermophilus* remains the only known non-pathogenic member of this genus, being a member of the salivarius group of the viridans streptococci. The origins of this species are somewhat controversial, but it seems likely that it has emerged through a combination of genome decay of other viridans streptococcal species and horizontal gene transfer events (Delorme 2008). While strains of the species are inextricably linked to fermented dairy foods, recent studies have demonstrated their ecological association with plant material (Umamaheswari et al. 2014). This is consistent with a traditional Bulgarian approach to yoghurt production in which the branch of a native plant was used to inoculate boiled sheep’s milk (Michaylova et al. 2007).

The identification of *S. thermophilus* and *Lactobacillus bulgaricus* in 1905 by the Bulgarian physician and microbiologist, Stamen Grigorov, incited scrutiny into the functions, activities, and metabolites of so-called ‘beneficial microbes’ and formed the foundations for the field of probiotic research (Lilly and Stillwell 1965). *Streptococcus thermophilus* is associated with the alleviation of lactose intolerance—the only probiotic claim that is currently recognized and accepted by regulatory bodies [Regulation (EC) No. 1924/2006]. Furthermore, its long history of safe application in food fermentations and human consumption are among the major factors underpinning its Generally Regarded as Safe status as recognized by the U.S. Food and Drug Administration and its inclusion in the Qualified Presumption of Safety list in the EU [EFSA Panel on Biological Hazards (BIOHAZ) et al. 2022].

The complete genomes of 83 *S. thermophilus* strains are currently available in the NCBI database (search date: November 1, 2022). The genomes of these strains are typically between 1.73 and 2.10 Mb (Table S1) and have an average of 39% G + C content. Recently, it has been proposed that there are two major clusters of *S. thermophilus* genomes (A and B) that may be differentiated based on gene gain or loss (Alexandraki et al. 2019). These two clusters can primarily be distinguished based on size, with Cluster A strains possessing genomes >1.83 Mb and Cluster B strains with genomes below this threshold value. Of the 83 genomes currently available in public databases, 55 would be classified as Cluster A and 28 as Cluster B genotypes based on the genome size criterion (Table S1). This is inkeeping with the findings of the comparison of 23 *S. thermophilus* genomes, in which ∼30% of strains were identified to have genomes of <1.83 Mb (Alexandraki et al. 2019). Among these 23 analysed genomes, the percentage of pseudogenes was in the range of ∼9% to 14%. While it is suggested that the pan-genome of this species may soon be closed, there are genomic regions of variability that provide elasticity to strains of this species and constant evolution in response to the pressures that are present in their natural or industrial environs. Among these regions of genetic variability are those relating to the biosynthesis of exopolysaccharides (EPS), rhamnose-glucose polysaccharides (RGP), and the CRISPR-Cas loci. In this review, we discuss recent advances in defining the role of cell wall polysaccharides in host recognition by phages and/or phage exclusion. Furthermore, we provide detailed insights into the core functions associated with the biosynthesis of EPS and RGP and explore how the diversification of the gene clusters that encode these structures may influence the evolutionary pathway of phages infecting *S. thermophilus*.

## EPS produced by *S. thermophilus*

The global starter culture market is currently valued at ∼$1.1 billion USD and is expected to increase to $1.5 billion USD by 2027 according to market research sources (BusinessWire, report, October 2022). Among bacterial starter cultures, *S. thermophilus* is the second most widely exploited species applied in food fermentations (after *Lactococcus lactis/cremoris*); however, recent growth in demand for plant-based alternatives to dairy fermented products may reverse this order in the coming decades (Harper et al. 2022). To illustrate this, *S. thermophilus* is currently applied for the production of a range of yoghurt-like products based on plant-based substrates, including oat, soy, almond, and cashew, among others (Montemurro et al. 2021). The primary function underpinning its widespread application in these products is the ability of many strains of *S. thermophilus* to produce exopolysaccharide (EPS), which contributes to the texture and mouthfeel of the product. The yield of EPS from *S. thermophilus* strains ranges from 20 to 600 mg/L (Vaningelgem et al. 2004), values that can be influenced by culturing conditions, carbon source, and co-cultivation with (non-EPS-producing) strain(s) of the same or other bacterial or yeast species (Zisu and Shah 2003, Sørensen et al. 2022). Interestingly, the composition or combination of EPS types is a major contributors to the rheology, texture, and microstructural properties of yoghurt, rather than simply the amount of EPS that is produced (Folkenberg et al. 2006).

In addition to their technological properties, the EPSs of certain *S. thermophilus* strains have been demonstrated to facilitate bacterial adhesion to gastric mucosa and reduce the adhesion capacity of *Helicobacter pylori*, while they have also been reported to diminish the expression of pro-inflammatory markers (Marcial et al. 2017). Furthermore, the EPS of *S. thermophilus* ST538 was shown to enhance expression of interferon β, interleukin 6, and C-X-C motif chemokine 10 in response to activation of toll-like receptor 3 in porcine intestinal epitheliocytes, suggesting that the EPS of this strain contributes to defence against viral infections (Mizuno et al. 2020). The activities and applications of EPS produced by *S. thermophilus* are of continued interest to the ever-expanding probiotics market [for an extensive review on this topic, see (Sørensen et al. 2022)].

**Figure 1.**
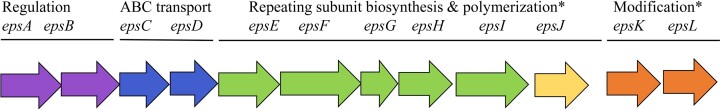
Schematic depicting the general architecture of *eps* gene clusters in *S. thermophilus* and highlighting some of the major functional units within these clusters, including regulation (purple arrows); transport and/or chain length determination (indigo arrows), repeating subunit synthesis, including glycosyltransferases (green arrows); and polymerization (yellow arrow); and modification functions (orange arrows). These gene clusters range in size from ∼15–30 kb. Note: *the nomenclature of the repeating subunit biosynthesis and modification genes may vary depending on the number of genes in an individual cluster, but are typically named sequentially.

The EPS structures produced by *S. thermophilus* strains are typically heteropolysaccharides containing glucose, galactose, rhamnose, and on occasion N-acetylglucosamine, galactosamine, and fucose (Bubb et al. 1997; Low et al. 1998; Ricciardi et al. 2002; Szymczak et al. 2018; McDonnell et al. 2020; Jurášková et al. 2022). The gene clusters that encode EPS biosynthetic functions vary extensively in size and genetic composition (Bourgoin et al. 1999; Parlindungan et al. 2022). Despite the sequence diversity of these gene clusters, there are core functions that are conserved among their associated gene products, for example, EPS chain length regulation, sugar transfer, subunit polymerization, and membrane translocation (Fig. 1) (Parlindungan et al. 2022). Functional analysis of core genes associated with these loci has provided insights into the mechanisms by which these structures are constructed and regulated. EPS production varies depending on the sugar substrate that is present in the medium, and *epsC* and *epsD* have been shown to regulate the chain length of the final EPS structure in ASCC 1275 (Padmanabhan et al. 2020). The gene products of *epsCD*, which are proposed to form a membrane-located complex, also bear topological and sequence similarities to ABC transport systems, suggesting that they play a dual role in EPS transport and chain length control (Stingele et al. 1996). It is assumed that the transcription level of *epsC* (and likely *epsD)* influences chain length, while the availability of nucleotide sugars may also modulate chain length also (Wang et al. 2020). Polymerization of the EPS repeating subunits is suggested to be a function of EpsJ, possibly through complexation with EpsC and EpsD (Stingele et al. 1996). Recent analyses of *eps* loci of dairy streptococcal isolates identified 10 distinct genotypes (named A–J), and the glycosyltransferase-associated gene content largely accounts for their differentiation (Szymczak et al. 2019, Romero et al. 2020, Parlindungan et al. 2022). Furthermore, the presence of transposase-encoding genes in many of these clusters may be associated with their diversification/mobilization within or between strains as well as their (lack of) functionality in certain strains (Parlindungan et al. 2022).

The diversity of the *eps* loci of dairy streptococcal strains is likely also linked to their compositional and structural diversity, with possible downstream impacts on their interactions with other microorganisms. However, while there is an ever-increasing number of genome sequences available as well as several studies detailing the EPS composition and/or structure, the links between these and the composition and structure remain poorly explored and represent a critical question that could be transformative to functional food development and human health alike. Furthermore, since certain dairy streptococcal phages are known to recognize and bind to EPS, it is essential to gain improved insights into the structural diversity of EPS that are produced by this species to identify their specific saccharidic receptors. This will improve risk evaluations by starter culture providers and ultimately facilitate increased consistency in dairy fermentations.

## RGP produced by *S. thermophilus*

In addition to EPS, all *S. thermophilus* strains produce a cell wall polysaccharide, termed the RGP, that is closely associated with the cell surface. The RGP of streptococci is implicated in interactions with other organisms, cell morphology, and cell division processes (De et al. 2017, Bischer et al. 2020, Lavelle et al. 2022). The designation of these polysaccharides as RGPs is an artefact of the nomenclature of similar polysaccharides in pathogenic streptococci, in which these structures were primarily observed to contain rhamnose and glucose (Mistou et al. 2016).

**Figure 2.**
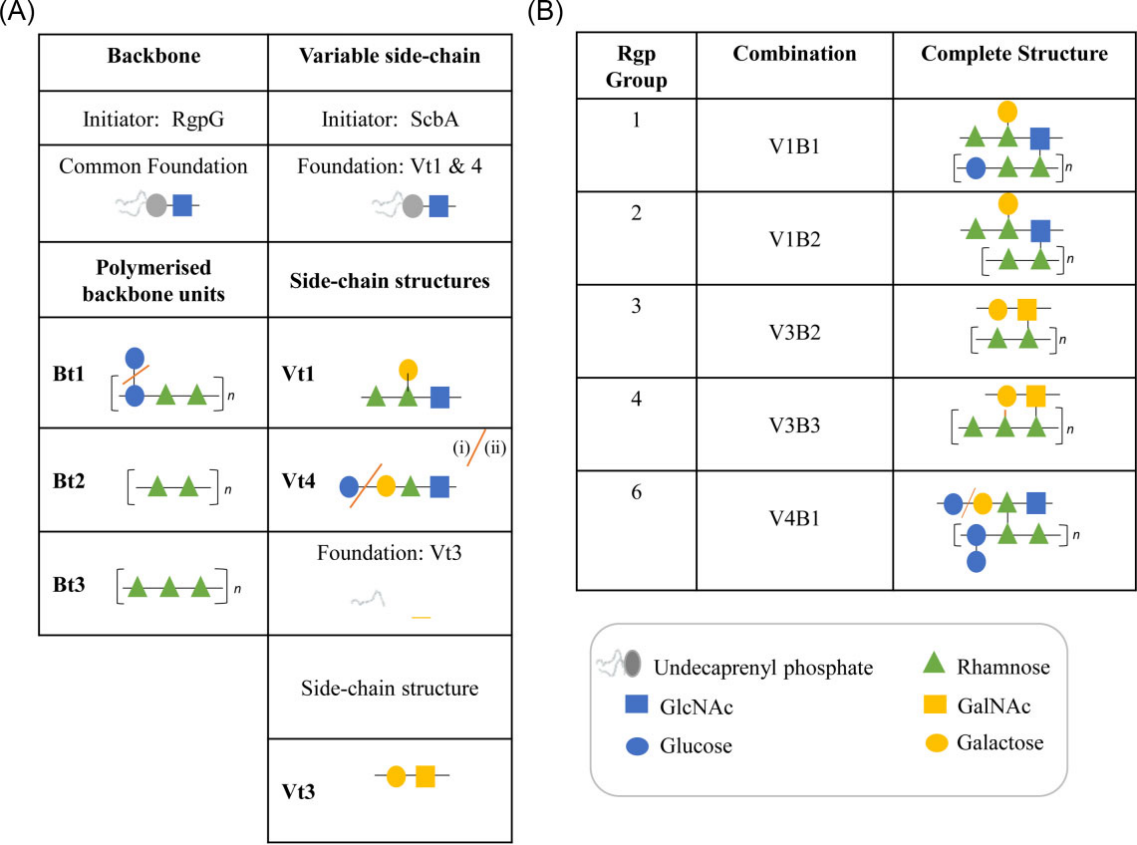
Panel **(A)** overview of the individual components of the *S. thermophilus* Rgp backbone **(B)** and variable side-chain (V) structures, which have been elucidated to date. Panel (B) distinct Rgp groups, their associated backbone-variable side-chain combination (VxBx), and the established biochemical structure. A red line indicates the following detected modifications: The Bt1 polymer may or may not carry a glucose modification; the Vt4 side-chain may be a tri- or tetra- saccharide; and the V3 side-chain of the Rgp4 structure may be attached to the polyrhamnose core at differing linkage points.

The RGPs of dairy streptococci have been found to contain N-acteyl glucosamine, N-acetyl galactosamine, or galactose in addition to rhamnose and/or glucose, with strain to strain variation in the monosaccharide composition, the number of monosaccharides in the repeating subunit, and the extent of branching of these structures (Szymczak et al. 2018, Szymczak et al. 2019, Romero et al. 2020, Lavelle et al. 2022, Lavelle et al. 2022, Parlindungan et al. 2022). The 20–30 kb gene cluster that encodes the biosynthetic machinery for the RGP structure, and which is termed the *rgp* locus is responsible for the synthesis of two connected saccharidic components. The first of these constitutes the backbone structure, which is believed to be covalently linked to and embedded within the peptidoglycan layer, while the second represents a side-chain structure which is covalently linked to the RGP backbone, is exposed at the cell surface, and has been recognized as the receptor for certain dairy streptococcal phages (Lavelle et al. 2022). Based on hierarchical clustering analysis of the protein complement encoded by 78 *S. thermophilus rgp* loci, seven *rgp* genotypes were identified (*rgp*1–7) and the corresponding RGP structure of strains representing five of these genotypes has been determined (Fig. 2) (Lavelle et al. 2022). The leftward end of the *rgp* locus is associated with the synthesis of the variable side-chain structure, while the rightward end of the cluster is associated with the synthesis of the backbone structure. While seven *rgp* genotypes (Rgp groups 1–7) have been discerned based on the overall gene content, these two distinct regions of the *rgp* gene cluster may be present in various combinations in different strains with three distinct backbone genotypes (Bt) and five variable side-chain (Vt) genotypes discerned to date based on detailed analyses of these clusters (Lavelle et al. 2022). A two-step multiplex PCR has been established to facilitate the rapid classification of dairy streptococcal strains based on their Bt and Vt genotypes, which can be applied for the identification of strains with novel of Bt and Vt combinations. It is proposed that the backbone part of the structure is embedded within the peptidoglycan layer while the variable side-chain is surface-exposed (Lavelle et al. 2022). Therefore, it is most likely that the surface-exposed side-chain is associated with phage binding. However, since only a small number of phage-host combinations have been studied in detail in this host species, it is important to evaluate the full extent of diversity of the RGP structures and the multiplex PCR is a useful tool to rapidly predict and discern such diversity.

Knowledge regarding the diversity of these *rgp* clusters and their associated RGP structures may serve as a basis for the prediction of strains with distinct cell wall polysaccharide compositions, while it will also facilitate rational starter strain uses. Such rational starter strain applications, whether they be through selected (phage-insensitive) strain blends and/or strain rotation regimes, are all intended to reduce the risk of bacteriophage proliferation in food fermentations.

While there is considerable and continually emerging sequence data for this species, we still lack insights into the combinations of *rgp* and *eps* loci in strains of this species. The combination of these loci and their encoded structures may help in the selection of strains for starter culture regimes and mixed cultures to ensure the stability of fermentations and to reduce the risk of phage predation and proliferation in a given fermentation facility. Insights such as these will expand the potential for functional and technological developments in sustainable food production systems.

## Bacteriophages of *S. thermophilus*

One of the most significant and persistent challenges to food fermentation is infection of starter cultures by bacteriophages (or phages). Phage infection of strains within a starter culture may impair growth and milk acidification rates and lead to product inconsistency, downgrading and, in severe cases, complete loss of product. Phages have been shown to be highly persistent in food production facilities over extended periods of time and, this is likely to be exacerbated by the repeated and intensive application of specific starter strains or starter culture blends and the aerosolization of phage particles (Rousseau and Moineau 2009; Verreault et al. 2011). Furthermore, phages infecting strains of *S. thermophilus* are widely reported to display a high tolerance to pasteurization and other thermal treatments while chemical sanitizers applied in dairy processing plants may reduce the phage load albeit in a phage- and sanitizer-dependent manner [for an extensive review on this subject, see (Marcó et al. 2019)]. However, research reports on these treatments are few and far between, and often lack a universal testing approach, e.g. testing in milk, whey, or rich medium backgrounds.

*Streptococcus thermophilus* infecting phages have recently been classified into five genetically distinct groups (Philippe et al. 2020; Hanemaaijer et al. 2021). Members of all five groups possess, long non-contractile tails and isometric capsids. The groups are termed the *Moineauvirus* (formerly termed the *cos* group)*, Brussowvirus* (formerly termed the *pac* group)*, Vansinderenvirus* (formerly termed the 5093 group), and 987 and P738 genera of the *Aliceevansviridae* family (formerly part of the *Siphoviridae* family). The Moineau- and Brussowviruses are the most prevalent in the industrial dairy fermentation context and, consequently, are the most intensely studied of the dairy streptococcal phages with respect to genome sequence analysis, genetic diversity, and interactions with their host (Romero et al. 2020). While all dairy streptococcal phages appear to have evolved from temperate ancestors, only (certain) members of the *Brussowvirus* genus are truly temperate phages (Neve et al. 2003; Arioli et al. 2018). Members of all other genera of dairy streptococcal phages are virulent. The genomes of dairy streptococcal phages are typically between 30 and 40 kb and exhibit a modular architecture with discrete modules containing genes encoding replication, morphogenesis, and lysis functions (and lysogeny-related functions in certain *Brussowvirus* phages) (Hanemaaijer et al. 2021). The genomes of these phages are highly plastic with significant evidence of recombination within, between, and beyond (dairy) streptococcal phage groups (Hanemaaijer et al. 2021).

Several phages capable of infecting *S. thermophilus* recognize and bind to either RGP or EPS components on the cell surface of the cognate host (Table 1) (Szymczak et al. 2018; McDonnell et al. 2020; Lavelle et al. 2022). Members of the *Brussowvirus* genus have been shown to bind to (part of) the RGP structure (Szymczak et al. 2018; Lavelle et al. 2022), while members of the 987 genus recognize and bind to EPS components (Szymczak et al. 2018; McDonnell et al. 2020). The receptors for these phages have been identified through genome sequence analysis of phage-resistant derivatives of the host strains and, in some cases, through complementation of the observed mutations *in trans* (Table 1). Furthermore, the host range of many phages is well established, and since the genomes of many of the available strains are sequenced, it is possible to link the phage-encoded receptor binding protein (RBP) sequence phylogeny to the host *eps/rgp* genotype based on hierarchical clustering data (Szymczak et al. 2019). Using such a comparative genomics approach to link the phylogeny of phage-encoded RBPs to host-encoded *rgp* and *eps* genotypes, it is proposed that *Moineauvirus* phage RBP phylogeny correlates with host *eps* genotypes, thus implicating EPS as the receptor of these phages, although this has not been experimentally validated (Szymczak et al. 2019). While there is emerging data regarding the target moiety of these phages, there remains a significant knowledge gap with respect to the specific EPS or RGP (oligo)saccharides that are recognized and bound by these phages. Furthermore, a model for the biosynthesis of dairy streptococcal RGPs has been proposed (Lavelle et al. 2022), and experimental confirmation of this process will be transformative in predicting the biological functions and chemical structures of uncharacterized and newly emerging strains.

**Table 1.** Summary of confirmed receptors of four individual dairy streptococcal phages.

Phage (genus)	Receptor	Reference
CHPC951 (*Brussowvirus*)	RGP	(Szymczak et al. 2018)
SW13 (*Brussowvirus*)	RGP	(Lavelle et al. 2022)
9871 (987)	EPS	(McDonnell et al. 2020)
CHPC926 (987)	EPS	(Szymczak et al. 2018)

The phage infection process commences with the binding of the phage to a cognate receptor on the cell surface and is mediated by the phage-encoded adhesion device, a multi-protein complex located at the distal end of the phage tail. A seminal study of dairy streptococcal phage-host interactions using the model phage DT1 20 years ago suggested that multiple phage proteins were involved in DT1-host interactions (Duplessis and Moineau 2001). This has been validated by recent analysis of a range of dairy streptococcal phages, in which multiple phage adhesion device proteins were shown to incorporate carbohydrate binding domains (CBDs) (Lavelle et al. 2020; Goulet et al. 2022). Adhesion devices typically incorporate (a portion of) the tail tape measure protein (TMP), distal tail protein (Dit), tail-associated lysin (Tal), RBP, and accessory proteins in some cases. Among these proteins, the Dit and Tal of several dairy streptococcal phages have been shown to incorporate extensions containing various CBDs. While, in principle, the RBP alone is sufficient to initiate interactions with the host, it is believed that the additional CBDs enhance the ability of the phage to gain proximity to its cognate host and to facilitate directed and specific contact between the RBP and the associated host-encoded receptor (Goulet et al. 2022). The interactions between dairy streptococcal phages and their hosts are highly specific, i.e. dairy streptococcal phages typically exhibit narrow host ranges, which is likely due to the diversity of the RGP and EPS structures that are presented on the cell surface. However, while binding to these structures represents the first step in the phage infection process, it is important to consider that the host presents a series of barriers to phage entry and proliferation that undoubtedly contribute additionally to the narrow host range of these phages.

Among the most significant developments in the field of phage-host interactions is the development of bioinformatics tools that provide detailed structure-function insights, including HHPred (Soding et al. 2005) and AlphaFold (Jumper et al. 2021). Structure predictions and domain searches using such resources have generated significant insights into the types of receptors that phages may recognise, as well as the possible conformations of the structures themselves, and the importance of such tools in this area is difficult to overstate. For example, in *S. thermophilus* phages, HHPred and AlphaFold2.0 identified the presence, type, and location of CBDs within adhesion device proteins, supporting the contention that protein-saccharidic interactions are employed by these phages (Lavelle et al. 2020, Goulet et al. 2022). Considering the alternative approach, i.e. X-ray crystallography, which can be challenging and time-consuming, the application of bioinformatic tools such as these is expected to be transformative to the field.

## Phage defences in dairy streptococci

### Restriction-modification and CRISPR–Cas systems: the primary defences

Dairy streptococci are heavily reliant on two phage defence systems i.e. clustered regularly interspaced palindromic repeat (CRISPR) systems [and the CRISPR-associated (*cas*) genes] and restriction and modification (R/M) systems. The first description of CRISPR–Cas systems of *S. thermophilus* was reported in 2005 (Bolotin et al. 2005). Following their identification in this species, a significant number of studies have reported on the diversity, functionality, and prevalence of dairy streptococcal CRISPR–Cas systems (Barrangou et al. 2007, Horvath et al. 2008). There are two classes of CRISPR–Cas systems that differ in having either a multi-Cas protein complex or a single multi-domain protein that binds to CRISPR RNA (Class 1 and 2, respectively) (Makarova et al. 2020). Class 1 systems are further divided into three types (I, III, and IV), for which there are nine, six, and three variants described, respectively (Makarova et al. 2020). Class 2 systems are divided into three types (II, V, and VI) with four, seventeen, and five variants of each type, respectively. Genomes of *S. thermophilus* strains have been reported to harbour up to four CRISPR–Cas loci (named CRISPR1–4), and among these, CRISPR1 and 3 are described as Type II-A systems and are the most prevalent and active systems in this species (Hao et al. 2018). CRISPR2 is classified as a Type III-A and appears to be a degenerate system that is non-functional in acquiring spacers in all strains studied to date. CRISPR4 is a member of the Type I–E systems and is found in a limited number of *S. thermophilus* genomes (Hao et al. 2018). Interestingly, it has been demonstrated that CRISPR–Cas systems are compatible with and complementary to R/M systems, and their combined activity increases phage resistance activity (Dupuis et al. 2013).

R/M systems are classified into four types (I–IV) depending on their subunit composition and architecture (Roberts et al. 2015). A recent study analysing the genomes of 23 *S. thermophilus* strains observed that type I systems were prevalent in the majority of strains with most harbouring at least one such system (Alexandraki et al. 2019). Several strains were also found to possess a type II system, and four of the 23 genomes harboured three such systems each. Eight of the 23 strains possess a type III R/M system, while almost half of the strains possess a type IV system (Alexandraki et al. 2019). Research pertaining to the activity of dairy streptococcal R/M systems is limited, although certain type II R/M systems have been demonstrated to be functional in this species (Guimont et al. 1993; Burrus et al. 2001; Dupuis et al. 2013).

Dairy streptococci do not typically harbour many (if any) plasmids. Since CRISPR–Cas systems are associated with the restriction of foreign DNA, including phage and/or plasmid DNA, the low abundance of plasmids in this species is likely attributable to the omnipresence of active CRISPR–Cas systems. A database (NCBI) search of complete genome sequences of 83 *S. thermophilus* strains suggests that 11 harbour at least one plasmid, and among these, three strains harbour two plasmids (Table S1). Lactococcal strains, on the other hand, typically harbour several plasmids and these are a rich source of diverse anti-phage defence systems, while the limited prevalence of plasmids in dairy streptococci would suggest that this species is not highly dependent on such mobile elements such as plasmids for phage defences. The identified dairy streptococcal plasmids range in size from 3.3–14.1 kb with the majority being ∼3.3–4.5 kb. The available annotations of these plasmid-related sequences do not provide significant functional information; however, several contain orphan type I R/M restriction, methyltransferase, or specificity subunits that may complement or expand the action of chromosomally encoded systems. In response to the R/M and CRISPR–Cas systems of dairy streptococci, phages may respond by acquiring methyltransferases and/or anti-CRISPR (Acr) encoding genes to circumvent the major obstacles presented by the host. To ascertain the prevalence and relatedness of streptococcal phage-encoded methyltransferases, the NCBI Virus (https://www.ncbi.nlm.nih.gov/labs/virus/vssi/#/) tool was used to interrogate both unclassified (but which includes members of the P738, 987, and *Vansinderenvirus* species) and classified *Siphoviridae, Brussowvirus*, and *Moineauvirus* members infecting *S. thermophilus*. A total of 55 proteins were returned using all available search criteria containing the terms ‘methyltransferase’ or ‘methylase’. These methyltransferases appear to belong to six distinct groups based on the phylogenetic tree output of the above-mentioned search. Methyltransferase-encoding genes were identified among members of the *Moineauvirus, Vansinderenvirus*, and 987 groups, but seemingly not among members of the *Brussowvirus* species. Moreover, certain phages harbour more than one methyltransferase, which is suggestive of regular exposure to (distinct) R/M systems (Table 2).

**Table 2.** Overview of abundance of methyltransferases and anti-CRISPR systems in dairy streptococcal phages.

Phage group	#Methyltransferases/#encoding phages	#Anti-CRISPR proteins/#encoding phages	#Phages encoding both
*Moineauvirus*	13/11	16/16	0
*Brussowvirus*	2/2	4/4	1
*Vansinderenvirus*	14/10	2/2	2
987	22/12	2/2	1
P738	0/0	0/0	0

Two Acr systems have been described in *S. thermophilus* phages, i.e. AcrIIA5 and AcrIIA6 (Hynes et al. 2018), and among these AcrIIA6 systems are reportedly present in 33% of evaluated virulent dairy streptococcal phage genomes. To ascertain the current prevalence and relatedness of streptococcal phage-encoded anti-CRISPR proteins, the NCBI Virus (https://www.ncbi.nlm.nih.gov/labs/virus/vssi/#/) tool was used to interrogate both unclassified and classified *Siphoviridae, Brussowvirus*, and *Moineauvirus* phages infecting *S. thermophilus*. A total of 24 proteins were returned using all available search criteria containing the search string ‘acr’. Three and eight phages were identified to harbour AcrIIA5 and AcrIIA6 systems, respectively, while a further 13 phages harbour so-called ‘Acr-like’ proteins based on the search terms used in this analysis. These systems are dominantly associated with *Moineauvirus* (16 phages) and *Brussowvirus* (four phages), while they also occur at a lower frequency among *Vansinderenvirus* and 987 phages (Table 2). Although the true prevalence may be greater (and limited by the quality of annotations), the presence of both methyltransferase and Acr systems highlights the common presence of such counter defences among streptococcal phages and their historic engagement with such defence systems. It is also noteworthy that certain phages possess both Acr and methyltransferases (Table 2). For example, the *Brussowvirus* TP-J34 is predicted to possess AcrIIA6 and a N-6 DNA methylase; the *Vansinderenvirus* SW19 is predicted to encode a SAM-dependent DNA methyltransferase and an ‘Acr-like’ protein; and the 987 phage SW16 is predicted to encode a site-specific DNA methyltransferase and an ‘Acr-like’ protein.

### Contribution of prophages to the *S. thermophilus* resistome

Prophages are known to prevent secondary infection by phages possessing a homologous repressor (Davies et al. 2016). It has long been suggested that the incidence of lysogeny among dairy streptococci is low. To corroborate this notion, the genomes of 83 strains of *S. thermophilus* that are deposited in the NCBI database and are defined as complete (as of November 1, 2022) were analysed for the presence of prophages using PHAST (Zhou et al. 2011). This prediction provides suggestions of possible ‘intact’, ‘questionable’ and ‘incomplete’ prophage-encompassing DNA regions. All evaluated genomes were predicted to possess at least one incomplete prophage region; however, manual inspection of the (highly) conserved ∼10 kb region deemed that it was unlikely to represent a phage based on BLASTn and Pfam analysis. Similarly, 18 genomes were suggested to harbour questionable prophage-encoding regions. While some of these contained genes that may be of phage origin, they appeared to represent orphan genes rather than cryptic or satellite prophage regions. Finally, the genomes of eight strains were predicted to harbour one intact prophage (thus representing ~9.5% of evaluated genomes) and among these (based on manual inspection and Pfam and BLASTn analysis), three appeared to be genuinely intact prophage regions with genome sizes of 47.8 and 48.7 kb (two strains), respectively (Table S1). However, although the remaining five predicted intact prophage genomes may not be complete, the presence of repressor-encoding genes may be associated with immunity against phages possessing homologous repressor-encoding genes (Johnson et al. 1981).

Beyond repressor-mediated immunity, certain prophages have been implicated in superinfection exclusion by heterologous phages via the small prophage-encoded lipoprotein *ltp* (Sun et al. 2006; Ali et al. 2014). Interestingly, when *ltp*_TP-J34_ was expressed in *Lactococcus*, it was observed to provide protection against the lactococcal *Skunavirus* P008 (Sun et al. 2006). Of note, phage escape mutants capable of bypassing Ltp possess deletions in the TMP-encoding gene (Bebeacua et al. 2013). In agreement with this finding, proteome analysis of purified phage particles of the mutant phages identified that the TMP was significantly smaller (66 kDa) relative to that of the parent phage (75 kDa) (Bebeacua et al. 2013). BLASTn searches for homologues of *ltp*_TP-J34_ identified eight such genes with >95% sequence identity over the full length of the gene (January 2023). These *ltp* homologues are present in the prophages of strains SK778 (TP-778 L), DSM 20617, ATCC 19258, NCTC 12958, and NWC_2_1, as well as in the genomes of phages VS-2018a and SW18. Recently, a non-inducible prophage of *S. thermophilus* M17PTZA496 was also demonstrated to contribute to the host strain's resistome through the presence of a cI-like repressor and *ltp* (da Silva Duarte et al. 2018). These findings support the hypothesis that cryptic prophages and orphan phage-derived genes may contribute to the fitness of the host.

While prophage carriage may present possible benefits, the fitness costs should also be considered. For example, prophage carriage in *S. thermophilus* DSM 20617 was shown to reduce the cell wall integrity and heat-resistance, while it was shown to simultaneously increase adhesion to solid surfaces, a trait that is linked to peptidoglycan breaks (Arioli et al. 2018). In addition to the virulent and lysogenic states, it is proposed that phages may be present in cultures as chronic infections or exist in the so-called ‘carrier state’ either attached to the external surface of cells or as internalized DNA (Somerville et al. 2022). In these states, phages challenge the kill-the-winner concept and may persist stably over long periods of time within cultures or factories. Indeed, closely related phages that infect lactic acid bacterial starter cultures have been observed over periods of up to a decade in Irish and Canadian cheese factories (Rousseau and Moineau 2009, Lavelle et al. 2018). This persistence behaviour may lend itself to the fluctuating selection dynamic, which occurs when the fitness costs of developing phage-resistance are unfavourable and leads to a (temporary) reduction in active phages against the host species/strain.

## Challenges in the development of robust starter cultures for application in industry

Despite extensive counteractive efforts in the dairy industry, phages continue to be a major problem in these fermentations. Consequently, food producers have incorporated several lines of defence to mitigate this risk, including improved sanitation regimes, air filtration, staff movement control, and starter culture optimization and rotation. In dairy streptococci, the development of bacteriophage-insensitive mutants (BIMs) has traditionally been achieved through exposure of the starter culture (or individual strains) to whey containing phages that are problematic against the culture or strain. The generation of spontaneous BIMs of *S. thermophilus* is largely facilitated by the innate CRISPR–Cas systems and has been the approach of choice by many starter culture providers for decades as it is inexpensive, requires limited expertize and facilities, it generally has a limited, if not completely negligible, impact on technological properties of strains, and is acceptable to regulatory authorities as a natural process (Mills et al. 2010; Chirico et al. 2014; Achigar et al. 2021). While this process allows companies to respond rapidly to emerging or persistent phage problems in a factory-specific manner, such CRISPR-derived BIMs may be rapidly overcome by evolved phages with single point mutations in the acquired spacer regions (which are typically ∼30 bp in length) (Deveau et al. 2008). Consequently, longer-term solutions are required to ensure the stability of fermentations, possibly in combination with the development of spontaneous BIMs.

Knowledge of the saccharidic receptors required by dairy streptococcal phages may guide starter culture providers to select streptococcal strains with distinct *rgp* and/or *eps* genotypes in starter culture blends or rotation strategies. This requires detailed knowledge of the phage types that are present in fermentation facilities and the exact culture composition and formulation that was applied in the factory. This represents a considerable challenge for several reasons and cannot be generalized since each factory will have (i) individual production and cleaning practices, (ii) phage testing methods and depth, (iii) distinct relationships with starter culture providers/ingredient suppliers, and (iv) different business organizations. Furthermore, this is a long-term approach that requires considerable investment to establish the prevalent phage(s) and suitable strains to replace the sensitive strain and/or a rotation strategy that will reduce the risk of phage proliferation where producers prefer to retain the application of a phage-sensitive strain. Therefore, it is imperative that producers are well informed by starter culture providers to ensure that good production practices are adhered to in order to reduce the risk of phage infection and proliferation in industrial fermentations.

It is most likely that every fermentation facility possesses resident phages that persist in the factory environment and that certain phages are also transiently present across the lifetime of a factory. Companies may wish to project that their phage problems are limited or even absent; however, a universal understanding and acceptance of the omnipresence of phages will enable the development of tailored solutions and facilitate risk reduction thereby ensuring the ongoing success of the sector. Historically, failed fermentations resulted in the disposal of large volumes of milk into effluent ponds, wastewater storage facilities, or it was applied on land or as feed to animals with considerable downstream environmental impacts (Campbell and Feldpausch 2022). Valorization of dairy waste and by-products is an emerging area of research and will have the dual impact of improving the sustainability of fermentation processes and removing waste and by-products from the production site to the valorization facility, with the downstream result of reducing phage populations in the production environs as well as the associated economic advantages (Russo et al. 2021, Naomi David et al. 2022, Carolin et al. 2023).

Reports of non-CRISPR mediated BIM development in *S. thermophilus* are limited, probably due to the success of CRISPR–Cas systems in the industrial context. This has created a void regarding alternative strategies to generating BIMs in this species. RGP-mediated BIMs typically exhibit growth impairment and are consequently less likely to be suitable for industrial application (Lavelle et al. 2022). An alternative to generating RGP-based BIMs is the incorporation of strains with distinct *rgp* genotypes. Since phages recognize specific saccharidic structures (associated with the *rgp* genotypes), strain blends or rotations based on different *rgp* genotypes reduces the risk of phage infection of more than one strain in that blend or rotation. Similarly, inclusion of diverse *eps* genotypes in a starter blend or rotation scheme could be applied to reduce the proliferation of phages that recognize EPS receptors. Knowledge of EPS diversity at both the genetic and structural level is limited to date and will likely be an area of research expansion in the coming years given its biotechnological and fundamental importance.

In dairy *Lactococcus* spp. conjugation of large plasmids that harbour phage-resistance systems, including abortive infection and R/M systems, is an approach that has been widely applied for the development of phage-resistant and robust cultures (Trotter et al. 2001, Fallico et al. 2012). As mentioned above, dairy streptococci harbour limited, if any, plasmids and those that are present are typically small and non-conjugative. This, combined with the presence of CRISPR-Cas and R/M systems, have limited the adaptation of *S. thermophilus* strains by conjugation. In contrast to lactococci, however, natural competence may be exploited in certain strains of *S. thermophilus* (Fontaine et al. 2010a) to facilitate the introduction of genetic material. A challenge associated with the application of this approach is the strain-specific nature of the competence phenomenon. Despite reports of improved methods to induce natural competence in dairy streptococci, it is not applied widely for the adaptation of dairy streptococcal strains (Fontaine et al. 2010b). Therefore, while there is significant potential for the development of robust starter cultures, some practical, regulatory, and bio(techno)logical barriers and bottlenecks need to be overcome in the coming decade to facilitate timely responses to fermented food producers’ needs.

## Conclusion

The starter culture industry is in a period of major transformation as consumer demand for fermented dairy products and dairy alternative products continues to increase. This diversification of products combined with increased production volumes emphasizes the need for starter culture providers to be able to rapidly identify strains with the appropriate technological properties, that can reduce phage proliferation issues and improve the predictability/reliability of food fermentation processes. Considerable advances have been made in our understanding of the diversity of *S. thermophilus*, its phages, and their interactome. However, as these advances have raised an equal or expanded number of questions, it is imperative that we develop a holistic view of dairy streptococci, the composition of their variable genome content and how this will shape the future of research in this species and its application in food fermentation. This should incorporate an overview of the combinations of *rgp* and *eps* genotypes that streptococci may possess in concert with the defence systems that they harbour to truly evaluate the various lines of defence that strains of this species employ and how these may be harnessed for the development of robust starter culture systems.

## Supplementary Material

fuad032_Supplemental_File
